# The Effect of Neck-Specific Exercise with or Without a Behavioral Approach in Chronic Whiplash-Associated Disorders: A Systematic Review and Meta-Analysis

**DOI:** 10.3390/muscles4040049

**Published:** 2025-10-27

**Authors:** Luís Correia, Paulo Carvalho, Luísa Amaral, Mário Esteves, Rui Vilarinho, Mariana Cervaens

**Affiliations:** 1 CIR, E2S, Polytechnic of Porto, Rua Dr. António Bernardino de Almeida, 4200-072 Porto, Portugal; luisdfcorreia@outlook.com (L.C.); ruivilarinho1@gmail.com (R.V.); 2 RISE-HEALTH, Center for Translational Health and Medical Biotechnology Research (TBIO), E2S, Polytechnic of Porto, Rua Dr. António Bernardino de Almeida, 4200-072 Porto, Portugal; paulocarvalho@ess.ipp.pt; 3 FP-I3ID, FP-BHS, Escola Superior de Saúde Fernando Pessoa, Rua Delfim Maia 334, 4200-256 Porto, Portugal; lamaral@ufp.edu.pt (L.A.); estevesm@ufp.edu.pt (M.E.); 4 RISE-HEALTH, Universidade Fernando Pessoa, Praça de 9 de Abril 349, 4249-004 Porto, Portugal

**Keywords:** neck-specific exercises, behavioral approach, whiplash, physiotherapy

## Abstract

Chronic whiplash-associated disorders describe a cluster of symptoms that result from a sudden neck acceleration/deceleration movement, including pain, musculoskeletal and neurological signs, inducing functional disability. The aim of this study was to compare the effects of physiotherapy treatment based on neck-specific exercises, with or without a behavioral approach, in individuals with whiplash-associated disorders. Computerized research was performed in PubMed, PEDro, National Institute for Health and Care Excellence, and ScienceDirect to identify randomized controlled trials that evaluated the effectiveness of neck-specific exercises, with or without a behavioral approach, for chronic whiplash. For the meta-analysis, the outcomes of pain and disability were assessed. Thirteen studies were included in the systematic review, with a total of 2427 participants of both sexes, with ages between 18 and 63 years. Although interventions with a behavioral approach decreased pain and disability more significantly in 4 and 6 studies when compared to neck-specific exercises without such an approach, respectively, the meta-analysis revealed no differences between them. Although interventions for chronic whiplash-associated disorders based on neck-specific exercises with a behavioral approach seem to be more effective in reducing pain and disability, there is no quantitative difference favoring one over the other.

## 1. Introduction

Whiplash-associated disorders (WADs) refer to injuries caused by sudden acceleration-deceleration movements [1]. While WAD is commonly used interchangeably with “whiplash,” it is important to note that whiplash specifically describes the injury mechanism, not the symptoms such as pain, stiffness, muscle spasms, and headaches, which occur without any visible lesions or structural damage. The prognosis of WAD is uncertain and varies, with some cases resolving acutely with full recovery, while others may develop into chronic conditions, leading to long-term pain and disability [2]. WADs are a widespread issue, with a notable increase in prevalence in recent years. A study conducted in Sweden estimates that this condition affects 325 per 100.000 adults [3]. European studies indicate that approximately 84% of individuals who experience a whiplash injury develop acute symptoms of whiplash-associated disorder within the first week, while about 38% continue to have chronic symptoms such as neck pain or headache at 12 months [4,5]. The Quebec Task Force has developed a classification system to categorize patients with WAD based on the severity of their symptoms. This system includes four grades: Grade 1, characterized by neck pain, stiffness, or tenderness; Grade 2, which involves musculoskeletal signs such as reduced range of motion and localized tenderness; Grade 3, marked by neurological symptoms like sensory deficits and muscle weakness; and Grade 4, which includes dislocation or fracture of the neck [6]. According to Chen et al. (2009), the injury mechanism in WAD occurs in three stages: In the first stage, both the upper and lower spine flex, resulting in the loss of cervical lordosis; in the second stage, the lower vertebrae extend, which gradually causes extension in the upper vertebrae, leading the cervical spine to form an “S”-shaped curve. During this phase, the lower vertebrae experience an extension force, while the upper levels are subjected to a flexion force; in the final stage, the cervical spine reaches full extension, accompanied by a shear force that compresses the posterior processes of the articular capsules of the vertebrae [7]. After whiplash trauma, nociceptive endings become more responsive, a phenomenon known as peripheral sensitization of nociceptors. This response serves as a protective mechanism to prevent further injury to the affected and surrounding tissues [8,9,10].

As such, physiotherapy interventions based on specific neck exercises, focusing on deep muscles, are considered a safe treatment for neck pain, with temporary and benign side effects associated with whiplash [11]. However, in cases where recovery does not fully occur, nociceptors continue to send nociceptive signals to the central nervous system, causing dorsal horn neurons to become hypersensitive. Neurotransmitters then trigger increased postsynaptic responses, leading to hyperexcitability and dysfunction of the central inhibitory pain pathways in the brainstem [10]. Therefore, given the significant and chronic nature of pain following whiplash injuries, behavioral therapy has been suggested to address treatment barriers, to improve adherence, and to prevent or manage the chronic pain that arises from this protective response. The result is a multidisciplinary approach involving cognitive, behavioral, and physical therapy, including neck exercises, which help increase pain tolerance, improve pain-related behavior, and promote symptom-free movement [12,13]. However, the available evidence in WAD patients is somewhat contradictory, with some studies demonstrating the beneficial effect of this multidisciplinary [11,14], and other studies showing no significant effect [15,16,17].

Therefore, considering this disparity and that there is no known meta-analysis on this topic, the present review aimed to systematize and quantify the existing evidence comparing the effects of a physiotherapy programme, based on specific neck exercises, with (NSEB) and without (NSE) a behavioral approach, in subjects with chronic grades 2 or 3 WAD.

## 2. Materials and Methods

### 2.1. Search Strategy and Eligibility Criteria

A literature search was carried out between March 2024 and August 2024, in the PEDro, Pubmed, National Institute for Health and Care Excellence (NICE), and ScienceDirect databases, to identify randomized controlled trials (RCTs) that evaluated the effectiveness of NSEB and NSE, in the treatment of chronic whiplash, published up to the date of the search. The search was carried out using keywords and medical subject headings (MeSH) terms associated with the following: Whiplash, Neck-Specific Exercises, and Neck-Specific Exercise, with the combination “Whiplash” AND “Neck-Specific Exercises”. In the PEDro database, the search was carried out with the following keywords: Whiplash and Neck-Specific Exercises. The defined inclusion criteria were as follows: RCTs that compared the effect of NSE and NSEB, in subjects diagnosed with chronic grades 2 or 3 WAD, articles published in Portuguese or English, classified ≥5 on the Physiotherapy Evidence Database scoring scale (PEDro). The defined exclusion criteria were systematic reviews, meta-analyses, undiagnosed neck pain, and studies without full access. To confirm these two criteria, review authors (L.C. and P.C.) read the abstracts of the articles, and, in case of doubt, the full text of all studies presented in the research was read and discussed between them. The information gathered by the researchers was reviewed by a third author (M.C.) to reach a consensus. This systematic review was registered in the International Prospective Register of Systematic Reviews (PROSPERO) (CRD42024566470) and was reported according to the Preferred Reporting Items for Systematic Reviews and Meta-Analyses Statement (PRISMA) Guidelines, to improve the presentation standards of systematic reviews and meta-analyses [18].

### 2.2. Data Extraction

Two review authors (L.C. and P.C.) independently extracted and compiled data from the included studies. A third reviewer (M.C.) resolved disagreements. For this review, the following information was taken from the articles: population, intervention, tests used for evaluation, and results.

### 2.3. Critical Appraisal

Risk of bias and methodological quality were assessed using the Cochrane risk-of-bias tool for randomized trials (RoB 2) and the PEDro scale, respectively. RoB2 is structured into five domains of possible bias, including possible bias in the randomization process (D1), possible bias in the intervention process (D2), possible bias due to lack of data on outcomes (D3), possible bias in the assessment of outcomes (D4), and possible bias in the formalization of results (D5). The overall risk of bias is classified into three categories: Low Risk of Bias (+), Some Concerns (!), or High Risk of Bias (−) [19]. The PEDro scale is a valid tool for assessing the methodological quality of clinical trials, based on the presence or absence of 11 criteria. The final classification results from the sum of the responses to criteria 2 to 11, with the value varying between 0 and 10 [17]. Based on the final score, the study can be classified as having poor quality (≤3), fair quality (4–5), good quality (6–8), or excellent quality (≥9) [20,21].

### 2.4. Statistical Analysis

In order to analyze the impact of NSE and NSEB in subjects diagnosed with grade 2 or 3 chronic WAD, the Review Manager (RevMan) software (version 5.4) was used. The Cochrane Collaboration 2020 was used to perform the meta-analysis. To carry it out, studies with similar characteristics that compared NSE with NSEB, using the same assessment instruments for the same outcomes at follow-up moments, were considered. For the selected RCTs and their continuous outcomes, the absolute differences between the intergroup mean values were measured using the mean difference (CI: 95%), based on the use of the same scale/unit. To assess the consistency of the results, statistical methods to measure heterogeneity were used, assessed by the Chi-Square (X^2^) test, proposed by Pearson, in order to detect significant heterogeneity, and through the I^2^ test, proposed by Higgins et al. (2021), to quantify the extent of this heterogeneity [22]. In order to describe significant heterogeneity and, due to the small number of RCTs analyzed, a cut-off value of 0.10 was considered for the *p* value in the X^2^ test. Regarding the extent of this heterogeneity through the I^2^ test, the presence of a range between 0 and 40% is not important, from 30 to 60% represents moderate heterogeneity, from 50 to 90% substantial heterogeneity, and, finally, a value within the range of 75 and 100% manifests a heterogeneous level. The analysis and relevance of the I^2^ test value must consider the magnitude and direction of the effect, as well as evidence of heterogeneity [19].

## 3. Results

### 3.1. Study Selection and Characteristics

**Figure 1:**
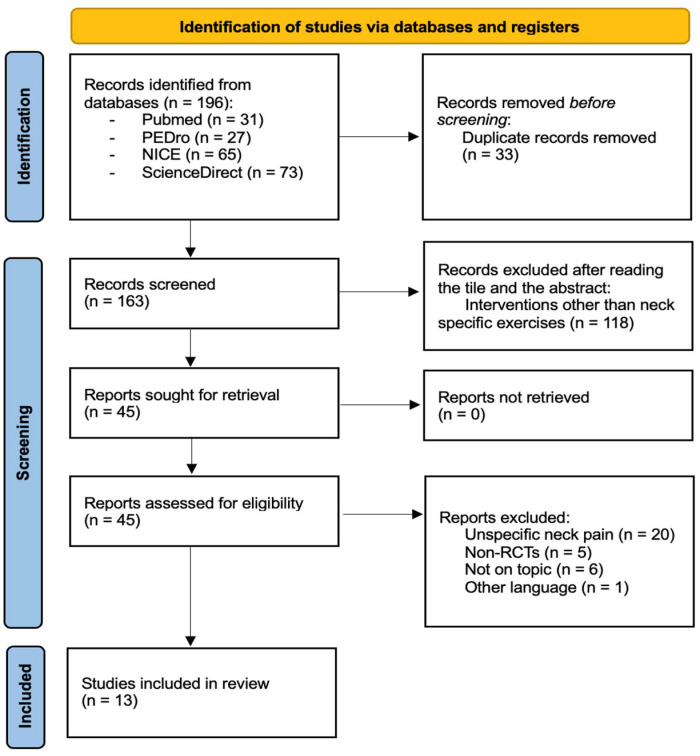
Flowchart of literature search.

A total of 13 studies were included in this review [11,14,16,17,23,24,25,26,27,28,29,30,31] (Figure 1).

The sample size ranged from 140 to 216 participants, 2427 in total, with a minimum age of 18 years and a maximum age of 63 years in all articles analyzed. In nine of the thirteen studies analyzed, 65% were female and 35% were male. There was great homogeneity regarding the type of intervention performed in the thirteen studies collected, which were the same type of treatment plan collected from a common source [27]. Regarding the type of intervention, it was performed by physiotherapists and divided into two groups. The first group, called NSE, included exercises focused on the deep cervical muscles, which were introduced with progressive resistance training of the head using a weighted pulley, together with home exercises focused on good posture and low load resistance. In this intervention group (IG), participants were educated not to enter the pain and to avoid reproducing symptoms of their clinical condition. Their intervention plan has the following structure: weeks 1, 2 and 3: Targeted exercises to facilitate the deep neck muscles through the use of isometric contractions (3–5 s contractions, 3 sets of 5 repetitions progressing to 3 sets of 10 repetitions); weeks 4 to 12: Specific cervical exercises in the gym using a weighted pulley with an initial load of 0.25 to 0.5 kg (3 sets of 5 repetitions progressing to 3 sets of 30 repetitions) and introduction of home exercises identical to those performed in the gym with low-load therabands. Regarding the NSEB group, the type of exercises and treatment plan were the same, excepting the introduction of coping strategies and the instructions given to the participants so that they performed the exercises in their full progression, even if this meant inducing pain or reproducing their clinical symptoms; therefore, in weeks 1, 2 and 3, coping strategies, postural control and relaxation techniques were introduced, and in weeks 4 to 12, the exercises were performed in the gym with attention to the gradual progression of the load, every two weeks. In all included studies, the participants of the control group (CG) received an individualized general physical activity prescription during these 12 weeks, without any specific exercises for the cervical spine. The number of treatment sessions was the same in all included studies, considering that they followed the same treatment plan. In each study, a total of 24 sessions were carried out for each intervention group, over a period of 3 months, twice a week. However, the interval between each treatment session was not specified. Table 1 shows a summary of the articles analyzed in this review with the characteristics of all participants and their assessment instruments, protocols, and results.

### 3.2. Risk of Bias and Methodological Quality

**Figure 2:**
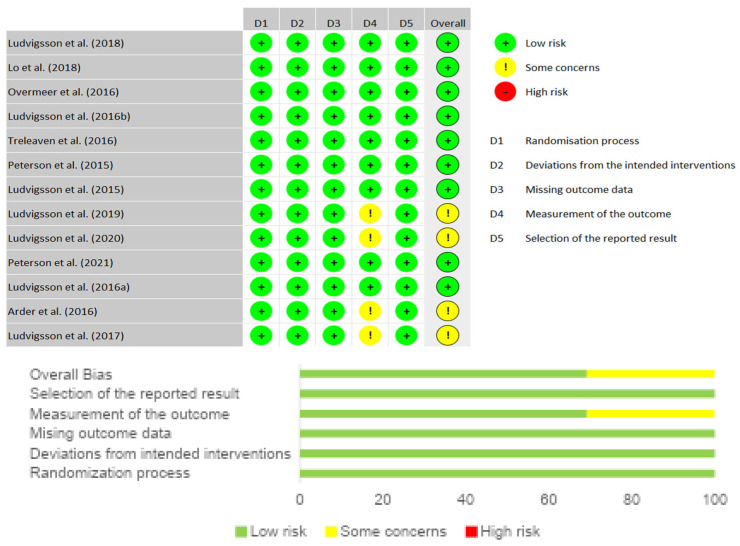
Summary plot and traffic light plot [11,14,16,17,24,25,26,27,28,29,30,31].

Based on the PEDro scale scores, the selected articles have an average classification of 6.9 out of 10, with a minimum score of 5 and a maximum of 8, thus having good scientific quality. RoB2 assessment is presented in Figure 2, by the inclusion of the Summary plot and Traffic Light Plot. Overall, nine studies were classified as “low risk of bias” and four studies as “some concerns”.

**Table 1:** Randomized controlled trials comparing Neck Specific Exercises (NSE) with Neck Specific Exercises with Behavioral approach (NSEB) in chronic Whiplash-associated disorders.

Study	Sample Characteristics	Aim and Duration of the Study	Outcomes	Results
Ludvigsson et al. (2018) [16] PEDro scale: 8/10	Age ≥ 18 and ≤63 years. Diagnosis of grades 2 or 3 WAD n = 171 participants. IG = NSE: 59 and NSEB: 59; CG = 53.	To examine two versions of specific cervical exercises or physical activity prescription in relieving brachial radiating pain and decreasing clinical signs associated with neurological deficits in people with WAD. Duration: 3 months.	Primary outcome: VAS for pain Secondary outcome: VAS for paresthesia Dermatome Myotomes Tendon reflex test Neurodynamic Test for median nerve	Improvements in the NSE and NSEB groups. With greater improvement in the NSE group after 3 months, VAS minimum pain (*p* = 0.01), VAS maximum pain (*p* = 0.01), VAS paresthesia (*p* = 0.11), Myotomes (*p* = 0.01), Dermatome (*p* = 0.04), Tendon reflexes test (*p* = 0.14), Neurodynamic Test (*p* = 0.26).
Lo et al. (2018) [11] PEDro scale: 7/10	Age ≥ 18 and ≤63 years. Diagnosis of grades 2 or 3 WAD n = 165 participants: IG = NSE: 60 and NSEB: 57; CG = 48.	To compare the effectiveness of two cervical-specific exercise interventions with and without a behavioral approach or physical activity prescription on self-reported work capacity for individuals with WAD Duration: 12 months	Primary outcome: WAI Secondary outcome: VAS for pain	Significant improvements in the NSEB Group in the WAI assessment, after 3 months (*p* = 0.03), after 6 months (*p* = 0.01), and after 12 months (*p* = 0.01) of treatment.
Overmeer et al. (2016) [24] PEDro scale: 8/10	Age ≥ 18 and ≤63 years. Chronic diagnosis of grades 2 or 3 WAD n = 194 participants: IG = NSE: 67 and NSEB: 68; CG = 59.	To investigate the effect of prescribing specific cervical exercises with or without a behavioral approach and prescribed physical activity on general pain disability and psychological factors in WAD patients Duration: 24 months.	Primary outcome: PDI Secondary outcome: PCS HAD TSK	Improvements in the NSE and NSEB groups, with greater improvement in the NSEB group, in the reduction in pain and disability after 24 months; PDI (*p* < 0.01); PCS (*p* < 0.01). Better results in the psychological factors of the NSE group, during the face-to-face follow-up after 12 months; HAD (*p* < 0.01) and TSK (*p* = 0.001).
Ludvigsson et al. (2016b) [25] PEDro scale: 7/10	Age ≥ 18 and ≤63 years. Chronic diagnosis of grades 2 or 3 WAD n = 202 participants: IG = NSE: 70 and NSEB: 68; CG = 64.	To compare, after 1 and 2 years, the effectiveness of two specific exercise interventions for the cervical spine with and without a behavioral approach or prescription of physical activity in reducing pain and increasing functional capacity in WAD. Duration: 24 months.	Primary outcome: NDI Secondary outcome: PSFS VAS for pain SES	Identical improvements in the NSE and NSEB groups, with greater improvement in NSEB, in the measurement of the primary outcome; NDI (*p* < 0.001). However, better results in NSE in the measurement; PSFS (*p* < 0.001); VAS (*p* < 0.001) and SES (*p* = 0.02).
Treleaven et al. (2016) [14] PEDro scale: 7/10	Age ≥ 18 and ≤63 years. Chronic diagnosis of grades 2 or 3 WAD n = 140 participants: IG = NSE: 41 and NSEB: 44; CG = 55.	To compare the effects of two specific cervical exercise interventions with and without a behavioral approach or prescription of physical activity on balance, dizziness, and proprioception in patients with WAD Duration: 12 months.	Primary outcome: VAS for dizziness UCLA-DQ Romberg Test Figure 8 walk HRA Secondary outcome: NDI VAS for pain	The results demonstrated significant improvements in the NSEB Group: VAS for dizziness (*p* < 0.01); UCLA-DQ (*p* < 0.001); Figure 8 walk (*p* < 0.001); HRA (*p* < 0.003); NDI (*p* = 0.02); VAS for pain (*p* < 0.01). Romberg Test without significant change between groups.
Peterson et al. (2015) [26] PEDro scale: 8/10	Age ≥ 18 and ≤63 years. Chronic diagnosis of grades 2 or 3 WAD n = 202 participants: IG = NSE: 70 and NSEB: 68; CG = 64.	To compare the effects of a cervical-specific exercise intervention with and without the addition of a behavioral approach to that of a general exercise intervention in patients with WAD Duration: 6 months.	Primary outcome: NME Secondary outcome: VAS for pain TSK Likert Scale	The results demonstrated significant improvements in the NSE Group: Ventral NME (*p* < 0.01); Dorsal NME (*p* < 0.01); VAS (*p* = 0.04); TSK (*p* < 0.01); Likert Scale (*p* < 0.01).
Ludvigsson et al. (2015) [27] PEDro scale: 8/10	Age ≥ 18 and ≤63 years. Chronic diagnosis of grades 2 or 3 WAD n = 216 participants: IG = NSE: 76 and NSEB: 71; CG = 69.	To compare the effectiveness of two specific cervical exercise interventions with and without a behavioral approach or physical activity prescription in reducing pain and disability in patients with WAD Duration: 6 months.	Primary outcome: NDI Secondary outcome: VAS for pain SES	The results demonstrated identical improvements in the NSE and NSEB groups: VAS (*p* < 0.001). With greater improvement in NSEB, in the measurement of the primary outcome; NDI (*p* < 0.001). Better results in (NSE) in the measurement; SES (*p* = 0.02).
Ludvigsson et al. (2019) [28] PEDro scale: 5/10	Age ≥ 18 and ≤63 years. Chronic diagnosis of grades 2 or 3 WAD n = 162 participants: IG = NSE: 54 and NSEB: 58; CG = 50.	To evaluate whether specific cervical exercise, with or without a behavioral approach, improves health-related quality of life (HRQoL) compared to physical activity prescription. Duration: 12 months.	Primary outcome: EQ-5D Secondary Outcome: EQ-VAS SF-36 PCS SF-36 MCS	Significant improvements in the NSEB group: EQ-5D (*p* < 0.01); EQ-VAS (*p* < 0.01); SF-36 PCS (*p* < 0.01); SF-36 MCS (*p* < 0.01).
Ludvigsson et al. (2020) [23] PEDro scale: 7/10	Age ≥ 18 and ≤63 years. Chronic diagnosis of grades 2 or 3 WAD n = 171 participants: IG = NSE: 59 and NSEB: 59; CG = 53.	To evaluate whether specific cervical exercise, with or without a behavioral approach, has long-term benefits over physical activity prescription in relation to arm pain and neurological deficits. Duration: 12 months.	Primary outcome: VAS for arm pain Secondary outcome: VAS for paresthesias Sensitivity test by dermatomes Muscle test by myotomes Tendon reflex test ULNT	Significant improvements for the NSE and NSEB groups, with greater improvement in the NSEB group after 12 months, VAS for pain (*p* < 0.01), VAS for paresthesias (*p* = 0.03), muscle test (*p* < 0.01), sensitivity test (*p* = 0.06) and ULNT (*p* = 0.02). Tendon reflex test did not show significant values between groups.
Peterson et al. (2021) [29] PEDro scale: 8/10	Age ≥ 18 and ≤63 years. Chronic diagnosis of grades 2 or 3 WAD. n = 160 participants: IG = NSE: 54 and NSEB: 59; CG = 47.	To evaluate whether specific cervical exercise, with or without a behavioral approach, improves clinical function and the relationship with self-reported functional disability. Duration: 12 months.	Primary outcome: NDI Secondary outcome: NME AROM Grip strength VAS for pain	The results showed significant improvements in the NSE and NSEB groups, with improvement after 12 months, NDI (*p* < 0.01), NME (*p* < 0.01), AROM (*p* < 0.01), grip strength (*p* < 0.01), VAS for pain (*p* < 0.01).
Ludvigsson et al. (2016a) [30] PEDro scale: 5/10	Age ≥ 18 and ≤63 years. Chronic diagnosis of grades 2 or 3 WAD n = 202 participants: IG = NSE: 70 and NSEB: 68; CG = 64.	To evaluate the effectiveness of specific cervical exercise, with or without a behavioral approach, in reducing disability and/or pain. To present long-term results after a short-term intervention. Duration: 12 months.	Primary Outcome: NDI Secondary Outcome: VAS	The results showed a significant improvement in terms of function in the NSEB group where NDI (*p* < 0.01), however in the outcome VAS for pain the NSE group (*p* = 0.01) presented a better result compared to NSEB (*p* = 0.04).
Arder et al. (2016) [17] PEDro scale: 6/10	Age ≥ 18 and ≤63 years. Have a chronic diagnosis of grades 2 or 3 WAD n = 168 participants: IG = NSE: 58 and NSEB: 57; CG = 53.	To determine the effectiveness of specific cervical exercises, with and without a behavioral approach, in increasing function and patient satisfaction among different types of intervention. Duration: 12 months.	Primary Outcome: Likert Scale (Satisfaction) Secondary Outcome: PEI	After 12 months there was a significant improvement in both intervention groups with regard to treatment satisfaction, with a slight improvement in the NSEB group where Likert Scale (*p* < 0.001) and PEI (*p* = 0.001).
Ludvigsson et al. (2017) [31] PEDro scale: 6/10	Age ≥ 18 and ≤63 years. Have a chronic diagnosis of grades 2 or 3 WAD n = 170 participants: IG = NSE: 58 and NSEB: 60; CG = 52.	To study the cost-effectiveness of using specific cervical exercises, with or without a behavioral approach, in improving quality of life. Duration: 12 months.	Primary outcome: ICER Secondary Outcome: EQ-5D SF-6D NDI	The NSEB group had a significant improvement in EQ-5D (*p* = 0.01), NDI (*p* = 0.001) and SF-6D (*p* = 0.07). The cost of the intervention was higher compared to the NSE group, which showed similar improvements in quality of life compared to CG, making this the best treatment for cost-effectiveness.

AROM: Active range of motion; CG: Control Group; EQ-5D: EuroQuol 5-dimension health questionnaire; EQ-VAS: EuroQuol Visual Analog Scale; IG: Intervention Groups; HAD: Hospital Anxiety and Depression scale; HRA: Head reposition accuracy; ICER: Incremental cost-effectiveness ratios; NDI: Neck Disability Index; NME: Ventral and Dorsal Neck muscle endurance; NSE: neck specific exercises without behavioral approach; NSEB: neck specific exercises with behavioral approach; PCS: Pain Catastrophizing Scale; PDI: Pain Disability Index; PEI: Patient Enablement Instrument; PSFS: Pain Specific Functional Scale; SES: Self-efficacy Scale; SF-36 MCS: Short-Form 36 Health Questionnaire Mental Component Summary; SF-36 PC: Short-Form 36 Health Questionnaire Physical Component Summary; SF-6D: Short Form 6-D health questionnaire; UCLA-DQ: University of California Los Angeles, Dizziness Questionnaire; TSK: Tampa Scale of Kinesiophobia; ULNT: Upper Limb Neural Tension Test; VAS: Visual Analog Scale; WAD: Whiplash-associated disorders; WAI: Work ability index.

### 3.3. Meta-Analysis

To perform the meta-analysis on the impact of NSEB or NSE, in subjects diagnosed with chronic grades 2 or 3 WAD, pain and disability outcomes were measured. In the analysis of pain, the Visual Analog Scale (VAS) was considered as the assessment tool, and data related to the 6 and 12-month follow-up were exported. Regarding the analysis of disability, it was evaluated by the Neck Disability Index (NDI), in an equal follow-up of 6 and 12 months.

#### 3.3.1. NSE vs. NSEB in Cervical Pain, Through VAS

**Figure 3:**
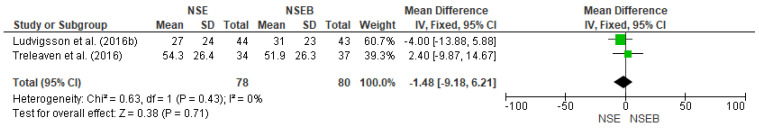
Forest Plot NSE vs. NSEB in neck pain after 6 months [14,25].

**Figure 4:**
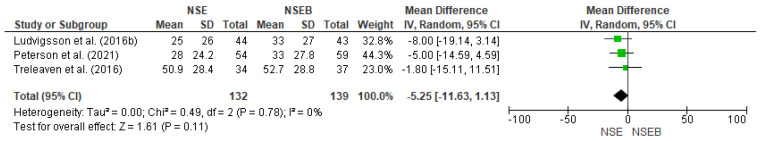
Forest Plot NSE vs. NSEB in neck pain after 12 months [14,25,29].

Of the eight studies that used VAS to classify neck pain, it was only possible to include three that met the criteria, with the follow-up being analyzed at 6 months in two of these studies, Ludvigsson et al. [25] and Treleaven et al. [14] (Figure 3), and at 12 months in the three studies [14,25,29] (Figure 4).

In a 6-month follow-up, using the VAS, it was found that there were no differences between the NSE and NSEB interventions in reducing neck pain (*p* = 0.71), with a null value of heterogeneity (I^2^ = 0%; *p* = 0.43), although a trend towards better results was noted after NSE. The same was true when pain was assessed after 12 months, in the three studies mentioned (Figure 4).

#### 3.3.2. NSE vs. NSEB on Cervical Functional Capacity Through NDI

**Figure 5:**
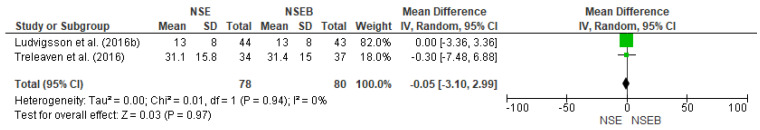
Forest Plot NSE vs. NSEB on functional capacity through NDI after 6 months [14,25].

**Figure 6:**
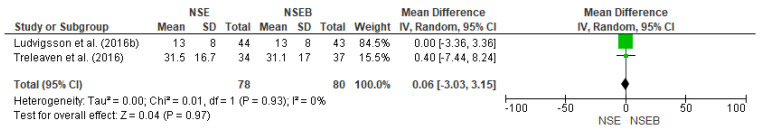
Forest Plot NSE vs. NSEB on functional capacity through NDI after 12 months [14,25].

Of the six studies that evaluated disability using the NDI, only two [14,25] met the criteria for analysis, and it was possible to assess the follow-up at 6 months and 12 months (Figure 5 and Figure 6, respectively).

Through the forest plot analysis, it was found that, in a 6-month follow-up, there were no differences between the NSE or NSEB protocols applied in functional capacity (*p* = 0.97), with a null value of heterogeneity (I^2^ = 0%; *p* = 0.94) (Figure 5). The same was true when functional capacity was assessed after 12 months, in the same studies (Figure 6).

## 4. Discussion

The aim of this systematic review with meta-analysis was to summarize and quantify the existing evidence comparing the effects of a physiotherapy treatment, based on NSEB or NSE, in subjects diagnosed with chronic grades 2 or 3 WAD. To date, this study stands out as a pioneer in analyzing the parameters in this condition, and it was officially registered in the PROSPERO database. In systematic reviews, assessing the risk of bias is an essential process to promote an accurate assessment of the general effects of the intervention process under analysis, and the RoB 2 scale was used for this purpose. In this review, most of the studies were found to be of low risk, with only four presenting some concerns regarding the assessment of outcomes [17,19,23,27]. The overall results suggest that NSE and NSEB are both effective interventions in reducing pain and disability.

### 4.1. Pain

In the studies [14,16,23,25,26,27,29], where VAS was used to measure pain symptoms, a significant improvement was observed in both NSEB and NSE, compared to the CG. Indeed, it was already established that pain is affected not only by tissue injury, but also by individual cognition, and there is evidence of evidence linking pain and disability to the patients’ cognitive response to pain experience [32]. Thus, although NSEB has been suggested to limit pain more significantly than exercise alone [33], the results of this meta-analysis did not corroborate such a hypothesis. The reasons why the NSEB group did not obtain better results in the present study could be related to several factors. First, the greater progression and intensity of exercise intervention in NSEB encouraged patients to perform the exercises in their full progression without considering pain threshold and the installation of their clinical symptoms. Second, the skills the patients acquired by performing only NSE could be helpful in the short and medium term, by improving muscle strength and postural stability, and, consequently, by modulating their pain levels experience [34]. Thus, the follow-up periods of 6 and 12 months might not be sufficient to reproduce all the benefits of NSEB interventions. Accordingly, the study of Overmeer et al. (2016) found a greater reduction in pain and disability in the NSEB group only after a 24-month follow-up [24]. In addition, a recent systematic review showed that, for chronic pain patients, a behavioral intervention may not always modulate pain intensity itself but can produce benefits regarding other aspects that impact patients’ pain perception, such as depression, anxiety, and quality of life (QoL) [35]. This was also noted in the studies of Ludvigsson et al. (2017; 2019), where the authors found a significant effect of NSEB on patients’ QoL and disability [28,31].

### 4.2. Disability

In the studies [11,14,17,24,25,29,30], it was observed that a significant long-term improvement in patients’ disability induced by NSEB, where participants, through coping methods and an education plan focused on exercise progression, led to a reduced disability and cervical kinesiophobia. However, when analyzing the forest plot of the meta-analysis carried out in the studies that could be included, there were no differences regarding disability between the NSE and NSEB at 6- and 12-month follow-ups. While the association between pain and disability is complex, it can lead to significant functional limitations, impacting the ability to carry on daily living activities. Therefore, considering the absence of effects observed in pain quantitative analysis, it was not surprising that the disability outcome also revealed no differences between the two interventions. Similarly, in the study by Sterling et al. (2014) [36], it was observed that exercise programs were effective in relieving the pain of WAD patients; however, these gains were not maintained in the long term, thus limiting their function. Therefore, it was suggested that education and pain management or physical exercise alone were not effective in reducing pain and improving functionality in individuals suffering from this chronic condition in the long term. Thus, a mixed approach including education but also advice about graduated functional exercises might induce greater improvements in pain intensity, pain discomfort, and functional capacity [36]. Accordingly, the study of Meeus et al. (2012) [12], found that an education program based on a behavioral approach combined with exercise therapy was more effective than oral education alone for patients suffering from disorders associated with chronic whiplash. However, several informational and educational approaches, including information leaflets, websites, and videos, have been investigated for their effectiveness in improving outcomes following whiplash injury. So, the great variability in the nature of the information and advice provided to a patient suggests that the best educational approaches, as well as strategies for behaviour change, have not yet been established to date. Moreover, considering that the concept of NSEB was introduced only in 2015 [27] to study the effectiveness of using an exercise program and coping strategies for pain and disability management, this impacts the evidence about the effects of such intervention in WAD patients.

### 4.3. Limitations

One of the main limitations encountered in conducting the present meta-analysis was the relatively small number of RCTs meeting the inclusion criteria. Several of these were continuations of previously conducted studies, while others lacked sufficient statistical data. Moreover, the available studies reported somewhat conflicting results, making it difficult to draw firm conclusions. Another important limitation is that a considerable proportion of the included studies originated from the same research groups, which may introduce bias and further restrict the generalizability of the findings. Since this is a topic only recently explored by the scientific community, consensus regarding the most effective approaches for treating disorders associated with chronic whiplash remains difficult to establish. Additionally, there was heterogeneity in the initial load prescribed when introducing the weighted pulley in the intervention protocols: some studies began with 0.25 kg, others with 0.5 kg, and in some cases with 1 kg (a load generally more suitable for men). Taken together, these factors highlight the need for caution when interpreting the present findings, and they underscore the necessity of further, more diversified studies comparing NSE and NSEB to better clarify which approach is most effective not only for managing pain and disability but also for other clinically relevant outcomes.

## 5. Conclusions

This systematic review with meta-analysis indicates that both NSE and NSEB might be helpful in reducing pain and disability in patients with grades 2 or 3 WAD. Nevertheless, because only a limited number of studies were included, these findings should be viewed cautiously, and it remains unclear whether one intervention can be considered preferable to the other. Additional high-quality research with larger sample sizes would be valuable to build a stronger evidence base and allow for more confident and generalizable recommendations.

## Data Availability

Not applicable.

## Abbreviations

The following abbreviations are used in this manuscript:

CG	Control Group
EQ-5D	EuroQol 5-dimension Health Questionnaire
EQ-VAS	EuroQol Visual Analog Scale
HADS	Hospital Anxiety and Depression Scale
HRA	Head Reposition Accuracy
ICER	Incremental Cost-Effectiveness Ratios
IG	Intervention Group
NDI	Neck Disability Index
NICE	National Institute for Health and Care Excellence
NME	Neck Muscle Endurance
NSE	Neck Specific Exercises
NSEB	Neck Specific Exercises with Behavioral approach
PCS	Pain Catastrophizing Scale
PDI	Pain Disability Index
PEDro	Physiotherapy Evidence Database Scoring Scale
PEI	Patient Enablement Instrument
PRISMA	Preferred Reporting Items for Systematic Reviews and Meta-Analyses Statement
PSFS	Pain Specific Functional Scale
SES	Self-efficacy Scale
SF-36-MCS	Short Form 36 Health Questionnaire Mental Component Summary
SF-36-PCS	Short Form 36 Health Questionnaire Physical Component Summary
SF-6D	Short Form 6-D health questionnaire
TSK	Tampa Scale of Kinesiophobia
UCLA-DQ	University of California, Los Angeles, Dizziness Questionnaire
ULNT	Upper Limb Neural Tension Test
VAS	Visual Analog Scale
WAD	Whiplash-Associated Disorders
WAI	Work Ability Index
